# Establishing the Most Appropriate Modified Atmosphere Composition to Store Dewi Star Fruit (*Averrhoa carambola* L.)

**DOI:** 10.1155/sci5/6694951

**Published:** 2025-09-25

**Authors:** Himawan Adinegoro, Lamhot Parulian Manalu, Ali Asgar, Nenie Yustiningsih, Wahyu Purwanto, Lanjar Sumarno, Henky Henanto, Subandrio, Edi Priyo Pramono, Amos Lukas, Rohmah Luthfiyanti, Siti Mariana Widayanti, Risfaheri, Agus Budiyanto, Abdullah Bin Arif

**Affiliations:** Research Center for Agroindustry, National Research and Innovation Agency, Jakarta, Indonesia

**Keywords:** packaging, postharvest, quality, senescence, storage

## Abstract

Star fruit is commonly cultivated in tropical regions, particularly in Southeast Asia, including Indonesia. This tropical fruit is known for its numerous beneficial nutrients and medicinal properties. However, star fruit is prone to damage and can rot quickly during storage. While several studies have explored the use of modified atmosphere packaging (MAP) for storing star fruit. However, a comprehensive study determining the optimal temperature, packaging, and atmospheric composition for storing star fruit is still limited. This study aims to design MAP for the storage of star fruit. The investigation focuses on how MAP affects changes in fruit quality during storage and how it can extend the shelf life of star fruit over a period of 40 days. The findings indicate that the senescence of star fruit is delayed when the fruit is packaged in polypropylene (PP) with a modified atmosphere consisting of 3% oxygen and 5%–7% carbon dioxide, all stored at 10°C and relative humidity (RH) of 80 ± 5%. This MAP treatment effectively reduces respiration rates and weight loss while preserving the fruit's firmness. The ideal plastic film for MAP at 10°C is PP, with a thickness of 0.039 mm and an average packaging surface area of 259 cm^2^. Each package contains one star fruit, averaging 281 g in weight. The combination of MAP and storage at 10°C significantly enhances the quality of star fruit and can extend its postharvest life by up to 40 days.

## 1. Introduction

Star fruit (*Averrhoa carambola* L.) is primarily cultivated in tropical regions, particularly in Southeast Asia, including Indonesia. As a tropical fruit, star fruit is known for its many beneficial nutrients and medicinal properties [1, 2]. It offers various health benefits, such as antioxidant, hypoglycemic, hypocholesterolemic, antihypertensive, anti-inflammatory, anti-infective, and antitumor effects, and immune-boosting capabilities [3]. Several studies have demonstrated that star fruit possesses antioxidant, anti-ulcer, anti-inflammatory, and antimicrobial properties [4, 5]. Additionally, it has positive effects on lipid status and glycemic control, exhibiting hypoglycemic activity [6, 7]. However, star fruit is prone to damage and rots quickly during storage, which presents a significant challenge. Maintaining its freshness and quality over an extended period is crucial. Therefore, effective postharvest technology is essential to preserve the quality and prolong the shelf life of star fruit.

Extending shelf life and preventing damage can be done by reducing the absorption rate of oxygen (O_2_) and releasing carbon dioxide (CO_2_) from star fruit. Packaging films with low permeability to oxygen and carbon dioxide to form a modified atmosphere is one technology that can be applied to overcome this problem in association with refrigeration. Modified atmosphere packaging (MAP) can reduce respiration, excessive transpiration, and enzymatic browning in horticultural commodities. Fang and Wakisaka [8] stated that MAP technology changes the gas composition in packaging suitable for storing horticultural commodities, such as nitrogen, oxygen, and carbon dioxide. It shows that the MAP technology maintains the quality and delays the senescence of horticultural commodities during storage.

The effect of low oxygen and high carbon dioxide in packaged atmospheric air can reduce the respiration rate and ethylene production rate and delay the deterioration of horticultural commodities [9]. This technology is seen as cheap and practical. This requires information regarding the optimum atmospheric composition and type of packaging film for each type of fruit and vegetable to achieve a longer shelf life. However, the standard determination of packaging type, oxygen composition, and carbon dioxide must be carried out precisely to achieve maximum effect. The appropriate packaging with optimal gas composition can extend the shelf life of vegetables and fruits and maintain their organoleptic qualities, such as taste, aroma, and color [10–16].

Several studies have examined the use of MAP for storing star fruit. Boonyaritthongchai and Kalayanarat [17] reported that passive MAP using polyethylene packaging can extend the shelf life of star fruit to 27 days when stored at a temperature of 10°C. Additionally, Chen et al. [18] found that a temperature of 5°C helps maintain the quality of “Honglong” star fruit, while storage temperatures of 0°C and 3°C cause severe chilling damage. Nimitkeatkai et al. [9] also noted that star fruit packed individually in low-density polyethylene (LDPE) bags effectively preserved its quality during storage. However, comprehensive studies determining the optimal temperature, packaging, and atmospheric composition for star fruit are still limited. Thus, establishing the proper standards for storage temperature, packaging type, oxygen composition, and carbon dioxide levels is crucial for improving the quality and shelf life of star fruit using modified atmosphere technology. This research aims to design effective MAP for star fruit storage. The expected benefit of this research is to identify optimal conditions that can be considered when designing packaging for long-term storage or transportation of fresh star fruit.

## 2. Materials and Method

### 2.1. Plant Material and Experimental Design

The current study involved the collection of star fruits from farmers' gardens in Tugu Village, Cimanggis District, Depok Regency, West Java, Indonesia. This location is situated at a longitude of 6.19° S and a latitude of 106.44°E, with an altitude of 140 m above sea level. The star fruit variety studied was cv. Dewi, which was harvested 70 days after the flowers bloomed, and was at the mature green stage. Cv. Dewi is a local cultivar with export potential. The collected fruits were transported to the Laboratory of the Research Center for Agroindustry at the National Research and Innovation Agency in Indonesia within 2 h. To ensure uniformity, the fruits were selected based on weight (200 ± 10 g), and only those without any physical defects were chosen. Each fruit was rinsed twice with tap water and then air-dried at room temperature.

### 2.2. Research Stages

#### 2.2.1. Determination of the Optimum Storage Temperature for Star Fruit Storing

A study on the optimal temperature for storing star fruit has been extensively conducted; however, there is limited information regarding the ideal storage temperature for the star fruit cv. Dewi. In this study, star fruit cv. Dewi was stored at four different temperatures: 5°C, 10°C, 15°C, and room temperature (26°C–27°C) and relative humidity (RH) (80 ± 5%) for three weeks (21 days). The experiment was conducted based on a factorial design. The first factor was temperature treatment, and the second factor was storage duration (0, 7, 14, and 21 days). A total of 204 fruits were used, with 17 star fruits allocated to each storage temperature and three replications for each condition. From each storage temperature group, 51 star fruits were selected to measure both destructive and nondestructive parameters on 36 and 15 fruits, respectively. Nondestructive measurements included the respiration rate (CO_2_ production and O_2_ consumption) and weight loss, while destructive measurements involved soluble solids content (SSC) and fruit firmness. All parameters were recorded every seven days during the 21-day storage period, in accordance with the measurement intervals established by Chen et al. [18] and Imahori et al. [19] on star fruit. This approach aims to determine the optimal storage temperature for star fruit.

##### 2.2.1.1. Measurement of Respiration Rate

The respiration rate was measured using a closed system; the star fruit was placed in an airtight glass container, and the O2 and CO2 concentrations were measured every 7 days for 21 days. The fruit was sealed in a 2.5 L gas-tight glass container. After 60 min, the O_2_ and CO_2_ contents were determined using an XPO 318 and XP 314 cosmotector analyzer. The respiration rate results were expressed as the CO_2_ released per second per 1 kg of fresh weight. Measurements were conducted at 5, 10, 15, and room temperature (26–27°C). The respiration rate was determined by linear regression of the relationship between changes in O_2_ and CO_2_ concentrations with storage time. The respiration rate was expressed as mL/kg·h.

##### 2.2.1.2. Measurement of Weight Loss

Weight loss is carried out using digital scales. Star fruit was measured for changes in initial weight until the weight decreased. The decrease in weight is carried out based on the percentage weight loss of the material from the initial day to the day of measurement [20].

##### 2.2.1.3. Measurement of SSC

The SSC was measured using the method suggested by Arif et al. [21] and Susanto et al. [22]. The SSC was measured from some extracted star fruit juice in triplicate using the Atago DR-A1 digital refractometer (Atago Co. Ltd., Tokyo, Japan) at a temperature of 28 ± 1°C. The SSC was expressed in °Brix.

##### 2.2.1.4. Measurement of Firmness

Fruit firmness was measured based on the fruit's resistance level to the piercing needle of a Shimadzu model CR 300 rheometer. The tool was set at a depth of 5 mm with a maximum load of 2 kg. The firmness measurement was carried out at three different points, namely at the base, the middle, and the tip of the fruit, with three repetitions, and then the average was taken.

#### 2.2.2. Determination of the Optimum Atmospheric Gas Composition for Star Fruit Storing

The optimum atmospheric gas composition was determined by experimenting with the effect of atmospheric composition treatment on the quality of star fruit during storage. The storage temperature for star fruit is based on the best storage temperature at the previous research stage. The experimental design was a completely randomized design, where the treatment was atmospheric composition consisting of 9 levels, namely 3% of O_2_ and 5% of CO_2_; 3% of O_2_ and 7% of CO_2_; 3% of O_2_ and 10% of CO_2_; 7% of O_2_ and 5% of CO_2_; 7% of O_2_ and 10% of CO_2_; 10% of O_2_ and 5% of CO_2_; 10% of O_2_ and 7% of CO_2_; 10% of O_2_ and 10% of CO_2_; and as control 21% of O_2_ and 0% of CO_2_ (ordinary air) (Table 1). The selection of gas composition is generally based on good tolerance conditions at 1%–5% O_2_ concentrations and 5%–10% CO_2_ [23]. The combination of atmospheric gas composition is regulated by regulating the O_2_ and CO_2_ gas using a MAP Mix 9001 ME machine (PBI Dansensor, Ringsted, Denmark). Each star fruit is packaged in polypropylene (PP) plastic packaging whose atmospheric air composition corresponds to 9 types of atmospheric air composition. The MAP Mix 9001 ME device draws all the gas in the packaging and is then filled with O_2_ and CO_2_ gas according to the composition of the atmospheric gas. A total of 135 star fruits were used at this stage. Each atmospheric gas composition treatment consisted of 15 units divided into three replications. Then, the star fruit was stored for 40 days (stored temperature based on the best temperature at the previous stage and RH [80 ± 5%]), and the parameters of fruit firmness, lightness, total sugar content, weight loss, and organoleptic assessment (firmness, sweetness, and color score) were measured. This stage aims to obtain the optimum atmospheric gas composition in maintaining the quality of star fruit cv. Dewi during storage.

##### 2.2.2.1. Measurement of Total Sugar

According to Ramadan et al. [24], total sugars were analyzed, where the anthrone reagent was 0.10% in concentrated sulfuric acid. The standard glucose solution is 0.20 mg/mL in 100 mL distilled water. Take 10 mL diluted to 100 mL (1 mL = 0.20 mg glucose). Pipette into test tubes blank 0, 0.20, 0.40, 0.60, 0.80, and 1 mL of standard glucose solution. Add distilled water to the total volume of each 1 mL test tube. Quickly add 5 mL of anthrone reagents to each test tube. Cover the test tube and shake it. Heat with boiling water for 12 min. Cool quickly using running water. Transfer to the cuvette and read the absorbance at *λ* = 630 nm. Make a curve of the relationship between absorbance and glucose concentration. Weigh the sample and add 100 mL of distilled water, strain with a filter cloth, then take 1 mL of the sample and dilute in 9 mL of distilled water until it is 100 times diluted.

##### 2.2.2.2. Measurement of Color

The color measurement uses a Minolta Chromameter type CR 310. Color data is expressed in L values (lightness), *a*^∗^ values (redness), and *b*^∗^ values (yellowness). The color measurement was carried out in three different parts of the star fruit (i.e., base, equatorial, and tip section). After that, the average was determined.

##### 2.2.2.3. Organoleptic Testing

Organoleptic tests were carried out to determine the panelists' level of acceptance of changes in the quality of star fruit during the experiment. Tests were conducted for hedonic scores for color, taste, and firmness parameters. The hedonic score used is on a scale of 1–5, where the score is 0 (*immensely dislike*), 1 (*dislike*), 3 (*regular*), 5 (*like*), or 7 (*like very much*).

#### 2.2.3. Determination of Packaging for Star Fruit Storing

The type of packaging film was determined after determining the optimum atmospheric gas composition (combination of O_2_ and CO_2_). In addition to using certain types of plastic films, other types with different permeabilities were also compared. Determining the type of packaging aims to select appropriate packaging to extend the shelf life of star fruit. The selection of plastic film for packaging was obtained from the graph, namely the line that cuts the optimum modified atmosphere area. The equation was plotted on a graph of the relationship between X_1_ (the O_2_ concentration in packaging) and X_2_ (the CO_2_ concentration in packaging). The film packaging chosen has lines cut through the modified atmosphere area. The type of film packaging selected can be seen in Table 2. The parameters measured include O_2_ and CO_2_ concentrations.

In addition, the MAP used was an active MAP, where the desired balance of O_2_ and CO_2_ composition was controlled at the beginning of packaging. Then, the balance of gas composition adjusts the permeability of the packaging film used while the star fruit is stored. For packaging validation tests, packaging design was previously carried out. In packaging design, the size of the packaging container used and the optimum weight of star fruit packed in each packaging were required. Determination of fruit weight and packaging area was carried out using the following calculation formula [26]:

(1) $$\begin{array}{c} X_{2}=C_{2}+\left(1/\beta \right)\left(C_{1}-X_{1}\right)\left(\frac{R_{1}}{R_{2}}\right), \end{array}$$

(2) $$\begin{array}{c} {\text{WR}}_{1}=\frac{P_{1}A\left(C_{1}-X_{1}\right)}{b}, \end{array}$$

(3) $$\begin{array}{c} {\text{WR}}_{2}=\frac{P_{2}A\left(X_{2}–C_{2}\right)}{b}, \end{array}$$

where *X*: gas concentration in packaging (%), *C*: ambient air gas concentration (%), *R*: respiration rate (ml/kg jam), *β*: CO_2_ and O_2_ permeability ratio of plastic film, W: fruit weight (kg), *A*: packaging surface area (m^2^), and *b*: thickness of packaging (mL). Indices 1 and 2 indicate O_2_ and CO_2_, respectively.

The data used in this equation were packaging thickness, permeability, area, and respiration rate to obtain the weight of star fruit packaged in a modified atmosphere.

### 2.3. Data Analysis

Data were analyzed with two-way analysis of variance at each storage period for stage (1). In addition, data were analyzed with a one-way analysis of variance at day 40 of storage for stage (2). The significant differences among the treatment means were determined by the least significant difference (LSD) at a probability level of 5%. Statistical analyses of data were performed by SAS Portable Version 9.1.3.

## 3. Result and Discussion

### 3.1. The Optimum Storage Temperature for Star Fruit Storing

The respiration rate of star fruit at different temperatures was measured to determine the optimal storage temperature for star fruit. A low respiration rate is usually accompanied by a long shelf life. Based on the respiration rate pattern, star fruit can be grouped into nonclimacteric fruit, and this shows that O_2_ consumption and CO_2_ production in star fruit during storage did not change significantly (Table 3). The average rate of O_2_ consumption was 1.60 mL/kg h, and the average rate of CO_2_ production in star fruit was around 1.58 mL/kg h (Table 3). Star fruit is reported to be a type of nonclimacteric fruit [18, 19, 27–30]. However, the observed behavior in O_2_ consumption and CO_2_ production of star fruit increased with increasing storage temperature (Table 3). Lameira et al. [31] reported that the respiration rate of star fruit stored at ambient temperature (25°C) was higher than at 5–10°C. In addition, Watson et al. [32] reported that storing fruits and vegetables at higher temperatures would increase respiration and thus accelerate senescence and deterioration. In this study, O_2_ consumption of star fruit during storage at 5°C and 10°C was not significantly different. Ali et al. [33] reported that storage at 10°C reduced water loss significantly, but at 5°C, water loss increased compared to that stored at 10°C, especially after day 10. Ali et al. [33] also reported that in addition to increased water loss, brown spots on the fruit surface, an early symptom of chilling injury (CI) in star fruit, also started to appear in fruit stored at 5°C for ten days.

As illustrated in Table 3, the weight loss rate of star fruit increased gradually at four different storage temperatures. After 21 days of storage, the weight loss of star fruit stored at 5°C and 10°C was minimal, ranging from 8.81% to 9.23%. In contrast, at room temperature, the weight loss reached 20.21% on day 21 of storage. This indicates that low-temperature storage effectively reduces weight loss in star fruit. According to Ali et al. [33], star fruit stored at room temperature experienced a nearly 20% loss in fresh weight by the end of a 3-week storage period. Firmness is a crucial indicator of fruit freshness, and the low-temperature treatments (5°C and 10°C) were more effective in maintaining the firmness of star fruit compared to storage at room temperature (26°C) over an extended period (Table 3). Additionally, the SSC of star fruit stored at low temperatures (5°C and 10°C) increased by 1%, from an initial 6.27% to 7.34% after 21 days of storage. On the other hand, the SSC of star fruit stored at room temperature increased by 2% during the same period (Table 3). This study reveals that low temperatures are more favorable for preserving fruit quality by slowing respiration rates, reducing weight loss, and maintaining firmness in star fruit during storage. Although there was no significant difference in the star fruit quality of storing at 5°C and 10°C, achieving the lower temperature of 5°C generally requires more cost and energy. Therefore, 10°C is the optimal storage temperature for star fruit.

### 3.2. The Optimum Atmospheric Gas Composition for Star Fruit Storing

A relatively slow softening rate characterizes star fruit ripening. One important physical parameter consumers use to assess postharvest fruit quality is firmness. As the fruit ripens, changes occur in the cell walls, leading to a decrease in both firmness and weight. To determine the optimal gas composition for a modified atmosphere that could extend the shelf life and maintain the quality of star fruit, experiments were conducted using various combinations of oxygen (O_2_) and carbon dioxide (CO_2_) treatments. The results revealed that the atmospheric gas composition significantly influenced the firmness and weight loss of star fruit during 40 days of storage (Tables 4 and 5). For example, the Dewi cultivar of star fruit, when stored under different atmospheric composition ratios (with 3% O_2_ and 5%–7% CO_2_) at a temperature of 10°C, exhibited a weight loss ranging from 37.1% to 37.3% by the 40^th^ day of storage. In contrast, without atmospheric modification (using 21% O_2_ and 0% CO_2_), the weight loss reached 68.4% by the 40^th^ day of storage. This indicates that the weight loss in star fruit with atmospheric modification was 32.1% lower than without atmospheric modification treatment. However, the atmospheric gas composition treatment did not significantly affect the fruit's color attributes—lightness, redness, yellowness, sweetness, or total sugar content (Tables 4 and 5). The sugar content of star fruit, essential for respiration during storage, decreased overall. In this study, the total sugar content across treatments remained relatively consistent at around 5%. As a nonclimacteric fruit, star fruit maintains a stable respiratory pattern, showing no spike in respiration during storage. Also, delaying color changes in star fruit is crucial for long-term storage.

The O_2_:CO_2_ composition treatment, namely 3:5 and 3:7, maintained fruit firmness and reduced weight loss compared to other gas composition treatments (Table 4). The packaging atmosphere modification treatment slows down the softening process and changes in the cell walls, in addition to further suppressing the activity of fruit wall enzymes [33]. MAP increases the shelf life of fruits by changing the microenvironment, thereby reducing respiration and increasing shelf life [34]. The main factors maintaining fruit quality in various atmospheric modification treatments are increasing CO_2_ and decreasing O_2_ levels, thereby reducing the respiration rate, preventing water loss, and increasing fruit firmness in strawberries [35]. When the amount of oxygen is reduced, there will be a delay in damage reactions (which may occur in the product), such as browning reactions and lipid oxidation, including the growth rate of spoilage organisms [36]. If the carbon dioxide level is more than 10%, it is phytotoxic to vegetables and fruit, and therefore, the CO_2_ concentration will be kept below this detrimental level. A delay in the respiration rate can suppress the cell wall degradation process so that it can maintain firmness and reduce weight loss in fruit [20–22]. The weight loss of star fruit in the MAP treatment was lower than in the control group; this was caused by the loss of ethylene and O_2_ in the headspace atmosphere [9]. It can catalyze enhanced respiration and transpiration [37–40]. Ali et al. [33] also suggested that the lower weight loss of star fruit packaged in MAP was caused by maintaining the structure of the membrane and cell walls. Therefore, the O_2_:CO_2_ gas composition treatment, namely 3:5 and 3:7, maintains the quality of star fruit during storage.

### 3.3. Types of Plastic Packaging and Star Fruit Weight in a Modified Atmosphere

**Figure 1:**
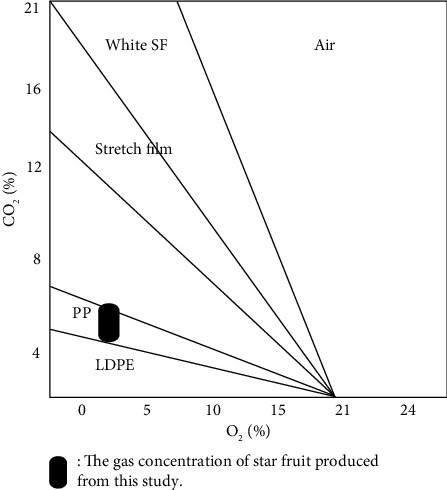
The optimum gas composition area for star fruit and the *β* line for several types of plastic.

Storing fruit in plastic packaging involves a dynamic process where the fruit continually uses oxygen for respiration, producing carbon dioxide (CO_2_) gas in the process. Due to the difference in oxygen (O_2_) levels inside and outside the packaging, oxygen enters the packaging while carbon dioxide is released [41]. According to Zagory and Kader [23], the oxygen content in packaging typically ranges from 2% to 5%, while the carbon dioxide content should not exceed 16%–19%, as higher levels can damage fresh produce. In previous studies, the optimal atmospheric gas composition for storing star fruit was found to be 3% oxygen and 5%–7% carbon dioxide. These gas compositions were chosen to evaluate the effectiveness of different packaging films. The optimal O_2_:CO_2_ ratio for storing star fruit is 1.67–2.33 (Figure 1). Plotting the selected atmospheric gas composition values on the film curve from Table 2, as in Figure 1, shows that the O_2_:CO_2_ (*β*) concentration ratio in PP packaging is 2.86, and in LDPE packaging, it is 3.59. Based on this curve, PP is selected as a suitable packaging film for storing star fruit because the gas concentration produced by star fruit with PP packaging is close to the optimum gas composition. Respiration rates that are too high can accelerate both transpiration and microbial growth. Conversely, low respiration rates, as observed in this study with LDPE packaging, inhibit the breakdown of polysaccharides into simple sugars. In contrast, the respiration rate of star fruit packaged in PP allows for a more optimal control of gas levels, making it a more suitable choice for packaging star fruit. It can be concluded that star fruit stored at a temperature of 10°C in PP packaging, with oxygen (O_2_) concentrations at 3% and carbon dioxide (CO_2_) between 5% and 7%, represents the best treatment for maintaining the quality of star fruit compared to other packaging.

In this study, packaging studies were carried out based on variations in the weight of the fruit being packaged (packaging containing one, two, and three pieces) and variations in the surface area of the packaging film. The results of equilibrium concentration measurements show that PP packaging containing one fruit reaches optimum storage conditions, namely 4% O_2_ and 6% CO_2_ (Table 6), where the equilibrium conditions are closest to the optimum atmospheric composition limits, namely 3% of O_2_ and 5%–7% of CO_2_. The packaging has a surface area of between 247 and 272 cm^2^ with an average of 259 cm^2^, and it contains 1 (one) star fruit that weighs between 268 and 301 g with an average of 281 g.

## 4. Conclusion

The study indicates that the senescence of star fruit can be delayed when the fruit is packaged in PP using a modified atmosphere that consists of 3% oxygen and 5%–7% carbon dioxide and stored at 10°C. This MAP effectively reduces the respiration rate and weight loss while maintaining the fruit's firmness. The optimal type of plastic film for MAP at 10°C is PP, with a thickness of 0.039 mm and an average packaging surface area of 259 cm^2^. Each package contains one star fruit with an average weight of 281 g. Overall, the combination of MAP and storage at 10°C significantly enhances the quality of star fruit and extends its postharvest life.

## Figures and Tables

**Table 1:** Atmospheric gas composition.

Atmosphere composition of O_2_:CO_2_ (%)	Concentration (%)	
	O_2_	CO_2_
3:5	3	5
3:7	3	7
3:10	3	10
7:5	7	5
7:10	7	10
10:5	10	5
10:7	10	7
10:10	10	10
21:0	21	0

**Table 2:** Permeability and ratio of O_2_:CO_2_ (*β*) of several types of plastic film.

Plastic film types	Thick (mm)	Water vapor permeability (g/m^2^/day)	Gas permeability (ml mil/m^2^·h)		Ratio of O_2_:CO_2_ (β)
			O_2_	CO_2_	
Polyvinyl chloride (PVC)	0.95	2.00	1033	389	0.38
Oriented polypropylene (OPP)	1.00	0.75	3007	1282	0.43
Low-density polyethylene (LDPE)	0.99	2.50	1002	3600	3.59
Polypropylene (PP)	0.61	1.00	229	656	2.86
Stretch film	0.57	6.50	4143	6226	1.50
White stretch film	0.58	4.00	1464	1470	1.00

*Note:* Source: Gunadnya [25].

**Table 3:** Effect of storage temperature on star fruit quality during storage.

Storage duration (d)	Storage temperature (°C)				Average
	5	10	15	26	
*CO*_2_ *production (mL/kg h)*					
0	1.82^a(A)^	2.83^c(A)^	4.56^b(A)^	14.55^a(AB)^	5.94^A^
7	1.36^c(B)^	2.87^c(A)^	3.74^b(B)^	14.16^a(B)^	5.53^B^
14	1.57^c(AB)^	2.59^c(B)^	4.56^b(A)^	13.85^a(B)^	5.64^B^
21	1.57^c(AB)^	2.65^c(B)^	3.96^b(B)^	15.46^a(A)^	5.91^A^
Average	1.58^c^	2.73^c^	4.20^b^	14.50^a^	

*O*_2_ *consumption (mL/kg h)*					
0	1.77^c(A)^	2.85^c(A)^	4.30^b(A)^	13.83^a(A)^	5.69^A^
7	1.20^c(B)^	2.73^c(A)^	3.47^b(B)^	12.52^a(B)^	4.98^B^
14	1.52^c(B)^	2.38^c(B)^	4.16^b(A)^	12.28^a(B)^	5.09^B^
21	1.90^c(A)^	2.36^c(B)^	3.63^b(B)^	14.84^a(A)^	5.68^A^
Average	1.68^c^	2.58^c^	3.89^b^	13.37^a^	

*Respiration quote (RQ)*					
	0.94^b^	1.06^a^	1.08^a^	1.08^a^	

*Weight loss (%)*					
0	0.00^a(D)^	0.00^a(D)^	0.00^a(D)^	0.00^a(D)^	0.00^D^
7	1.97^c(C)^	2.23^c(C)^	5.23^b(C)^	6.67^a(C)^	4.03^C^
14	4.98^c(B)^	5.27^c(B)^	10.21^b(B)^	12.87^a(B)^	8.33^B^
21	8.81^c(A)^	9.23^c(A)^	15.34^b(A)^	20.21^a(A)^	13.40^A^
Average	3.94^c^	4.18^c^	7.70^b^	9.94^a^	

*Firmness (kg/cm* ^2^)					
0	12.17^a(A)^	12.17^a(A)^	12.17^a(A)^	12.17^a(A)^	12.17^A^
7	11.65^a(B)^	11.47^a(B)^	11.27^b(B)^	11.08^b(B)^	11.37^B^
14	10.97^a(C)^	10.85^a(C)^	10.24^b(C)^	10.07^b(C)^	10.53^C^
21	10.54^a(D)^	10.21^a(D)^	9.87^b(D)^	9.67^b(D)^	10.07^D^
Average	11.33^a^	11.18^a^	10.89^b^	10.75^b^	

*Soluble solid content/SSC (°Brix)*					
0	6.27^a(B)^	6.27^a(C)^	6.27^a(C)^	6.27^a(C)^	6.27^D^
7	6.45^b(AB)^	6.54^b(BC)^	7.21^a(B)^	7.54^a(B)^	6.94^C^
14	6.73^b(AB)^	6.84^b(B)^	7.45^a(B)^	7.87^a(B)^	7.22^B^
21	7.26^b(A)^	7.34^b(A)^	8.12^a(A)^	8.31^a(A)^	7.76^A^
Average	6.68^b^	6.75^b^	7.26^a^	7.50^a^	

*Note:* Different lowercase letters in the same lines or different capital letters in the same column indicate a significant difference by the LSD test (*p* < 0.05).

**Table 4:** Effect of atmospheric gas composition on star fruit quality on 40 days of storage.

Atmosphere composition (% O_2_:CO_2_)	Firmness (kg/cm^2^)	Skin color			Weight loss (%)	Total sugar (%)
		*L*	*a*	*b*		
3:5	11.73^A^	19.23	−1.95	27.66	37.1^C^	5.10
3:7	11.71^A^	19.90	−2.01	27.89	37.3^C^	5.05
3:10	12.01^A^	18.96	−2.11	28.44	43.1^B^	5.06
7:5	11.65^AB^	19.82	−2.31	28.79	46.1^B^	5.47
7:10	9.65^ABC^	20.75	−2.09	28.31	40.2^B^	5.83
10:5	10.21^AB^	19.22	−1.98	28.88	43.3^B^	5.08
10:7	8.83^BC^	18.81	−2.05	29.01	43.4^B^	4.89
10:10	9.57^ABC^	19.76	−2.18	28.69	45.2^B^	4.92
21:0	7.35^C^	18.87	−2.83	29.87	68.4^A^	4.84

*Note:* Different capital letters in the same column indicate the significant difference by the LSD test (*p* < 0.05).

**Table 5:** Effect of atmospheric gas composition on consumer preferences of star fruit on 40 days of storage.

Atmosphere composition of O_2_:CO_2_ (%)	Score		
	Firmness	Sweetness	Color
3:5	5.6^A^	4.9	6.4^A^
3:7	5.4^A^	4.8	4.9^B^
3:10	5.3^A^	4.8	4.3^BC^
7:5	4.9^AB^	5.3	4.8^B^
7:10	5.1^A^	5.1	4.3^BC^
10:5	4.5^B^	5.2	4.9^B^
10:7	5.1^A^	5.0	4.9^B^
10:10	4.7^B^	4.8	4.8^B^
21:0	4.4^B^	5.3	4.0^C^

*Note:* Different capital letters in the same column indicate the significant difference by the LSD test (*p* < 0.05).

**Table 6:** The O_2_ and CO_2_ concentrations from calculations and determinations.

Fruit number per packaging	Total fruit weight (g)	Packaging area (cm^2)^	Calculations		Determinations	
			O_2_ (%)	CO_2_ (%)	O_2_ (%)	CO_2_ (%)
1	281	259	5	6	4	6
2	410	286	0	8	9	11
3	847	394	−10	12	13	16

## Data Availability

The data used during the current study are available from the corresponding author upon reasonable request.
